# Role of the short isoform of the progesterone receptor in breast cancer cell invasiveness at estrogen and progesterone levels in the pre- and post-menopausal ranges

**DOI:** 10.18632/oncotarget.5082

**Published:** 2015-08-24

**Authors:** Thomas McFall, Mugdha Patki, Rayna Rosati, Manohar Ratnam

**Affiliations:** ^1^ Barbara Ann Karmanos Cancer Institute and Department of Oncology, Wayne State University, Detroit, MI, USA

**Keywords:** breast cancer, progesterone receptor, estrogen receptor, invasiveness

## Abstract

Overexpression of the progesterone receptor (PR) isoform A (PR-A) is a negative prognosticator for estrogen receptor (ER)-positive breast cancer but *in vitro* studies have implicated PR-B in progestin-induced invasiveness. As estrogen is known to suppress invasiveness and tumor progression and as the *in vitro* studies were conducted in models that either lacked ER or excluded estrogen, we examined the role of PR isoforms in the context of estrogen signaling. Estrogen (< 0.01nM) strongly suppressed invasiveness in various ER+ model cell lines. At low (< 1nM) concentrations, progestins completely abrogated inhibition of invasiveness by estrogen. It was only in a higher (5 nM — 50 nM) concentration range that progestins induced invasiveness in the absence of estrogen. The ability of low dose progestins to rescue invasiveness from estrogen regulation was exclusively mediated by PR-A, whereas PR-B mediated the estrogen-independent component of progestin-induced invasiveness. Overexpression of PR-A lowered the progestin concentration needed to completely rescue invasiveness. Among estrogen-regulated genes, progestin/PR-A counter-regulated a distinctive subset, including breast tumor progression genes (e.g., HES1, PRKCH, ELF5, TM4SF1), leading to invasiveness. In this manner, at relatively low hormone concentrations (corresponding to follicular stage and post-menopausal breast tissue or plasma levels), progesterone influences breast cancer cell invasiveness by rescuing it from estrogen regulation via PR-A, whereas at higher concentrations the hormone also induces invasiveness independent of estrogen signaling, through PR-B. The findings point to a direct functional link between PR-A and progression of luminal breast cancer in the context of the entire range of pre- and post-menopausal plasma and breast tissue hormone levels.

## INTRODUCTION

The process of breast oncogenesis is believed to span up to several decades. Most (> 78 percent) of newly diagnosed breast cancer cases occur in women that are older than 50 years [1] and the median age at diagnosis is 61 years [2]. In most cases the tumors express the estrogen receptor (ER). ER+ tumors are exquisitely sensitive to anti-estrogen therapy. However, ER+ breast cancer is often metastatic at the time of diagnosis and metastatic ER+ tumors also frequently appear after many years of dormancy [3, 4]. In either case, the metastatic disease is generally incurable and even targeted therapies are generally only palliative. Therefore, it is necessary to understand more about deregulated molecular mechanisms that confer invasive properties on ER+ breast cancer cells. Clearly, both pre-menopausal and post-menopausal events that influence breast tumor invasiveness are clinically highly significant in breast tumor progression. Profound decreases in the levels of circulating estrogen and progesterone are a hallmark of post-menopausal physiology although, in post-menopausal women, breast, endometrial and adipose tissues contain much higher levels of estrogen and progesterone, compared to plasma levels of the hormones [5–9].

As the progesterone receptor (PR) gene is a target of estrogen, the PR expression status of ER+ breast tumors is believed to reflect the robustness of ER signaling and hence predict patient response to anti-estrogen therapy. Nevertheless, PR agonists do directly support invasiveness and metastatic potential in ER+/PR+ breast cancer cells as demonstrated using *in vivo* experimental models [10, 11]. The physiological relevance of these model systems is supported by the observation that in postmenopausal women, hormone replacement therapy with the combination of estrogen and progestin was associated with increased incidence of invasive breast cancer and breast cancer mortality compared with non-users [12] whereas estrogen monotherapy in women with prior hysterectomy was associated with a persistent decrease in the onset of invasive breast cancer [13]. However, in post-menopausal women who are not undergoing hormone replacement, the role of the endogenous hormones in the progression of ER+/PR+ breast tumors is unclear.

PR has two isoforms, A and B, that are expressed by alternative promoter usage from a single gene; PR-B is identical to PR-A except for the presence of an additional 164 amino acid amino-terminal segment that contains within it, an additional activation function, AF3 [14]. PR-B and PR-A exhibit both distinctive and overlapping patterns of agonist-induced gene activation or gene repression, depending on the variable contexts of the target promoters and the nature of the associated chromatin sites of PR binding [14–16]. In cells expressing equal amounts of PR-A and PR-B, a substantial proportion of the two proteins are sequestered by forming a heterodimer; the heterodimer regulates a smaller and unique set of genes compared to the homodimers [15, 17]. Clinical studies have shown that although in normal breast PR-A and PR-B are expressed at comparable levels, this balance is commonly altered during breast oncogenesis with a predominance of a high PR-A:PR-B ratio in early as well as progressed lesions [18]. An elevated PR-A:PR-B ratio, which is frequently due to overexpression of PR-A, is associated with a lower rate of disease free survival [19].

*In vitro* molecular studies have shown that when hormone-depleted breast cancer cells are treated with PR agonists, they induce invasiveness through several non-genomic and genomic signaling pathways of progestin [20–26]. Some of those studies have further reported that it is PR-B that mediates progestin-induced invasiveness *in vitro* [21, 27]. The progesterone doses that were used to demonstrate substantial PR-B dependent effects on invasiveness *in vitro* were relatively high, corresponding to the plasma range of the hormone levels associated with only the luteal phase of the menstrual cycle or with pregnancy. Horwitz and co-workers have also elegantly demonstrated *in vitro* that the mere overexpression of PR-A confers an inherently more aggressive phenotype in breast cancer cells, including adhesion to extracellular matrix, migratory capacity and survival, due to hormone-independent gene regulation by PR-A [28].

Most breast tumors are ER+ [29] and continue to retain ER expression even as they progress to hormone-independence [30, 31]. Estrogen supports the growth of ER+ breast tumors but it suppresses invasiveness of the tumor cells whether or not their growth is hormone-sensitive and also suppresses breast tumor progression [31–37]. However, *in vitro* studies of the role of PR in breast cancer cell invasiveness have generally been investigated mechanistically in the absence of estrogen signaling. The studies have either used ER+ cell line models in the absence of estrogen or they have relied on forced expression of PRs in ER-negative cells [21, 27, 38–40]. The relative contributions of PR ligands to invasiveness through opposing the suppressive effect of estrogen and the underlying mechanisms are still unclear in the literature.

Further, although the gene regulatory profile of ER has been shown to be estrogen dose-dependent [41], it is less clear whether PR has distinct mechanisms of action that depend on progesterone dose. The plasma levels of estrogen in pre-menopausal women is 1.4 nM–1.6 nM in the follicular phase and 3.6 nM–4.2 nM in the luteal phase [42]. Plasma levels of estrogen in post-menopausal women is 0.027 + 0.01 nM wheras breast tissue levels of estrogen in post-menopausal women is 1.4 + 0.7 nM [5, 6, 43]. The plasma level of progesterone ranges from 0.6 nM to 4 nM in the follicular phase and increases up to > 50 nM in the luteal phase [44] whereas post-menopausal women have a wide range of 0.047nM to 0.318nM (median 0.127 nM) [45]. The breast tissue level of progesterone in post-menopausal women is above an order of magnitude greater than its plasma levels [7]. Therefore, further investigation of the role of the individual PR isoforms on ER+ breast cancer cell invasiveness in the context of estrogen signaling and in the physiological range of breast tissue hormone levels was needed to more fully understand early events in hormonal regulation of breast cancer progression.

The ER+ model cell lines used in this study included T47D (ER+/PR+), ZR-75-1 (ER+/PR+) and BT474 (ER+/PR+/HER2+) cells. All three cell lines express both PR-A and PR-B. To dissect the actions of the individual PR isoforms, we also used recombinant T47D cells generated by Dr. Kathryn Horwitz and co-workers that virtually exclusively expressed PR-A or PR-B in addition to ER [46].

## RESULTS

### Estrogen dose dependence for inhibition of invasiveness

**Figure 1:**
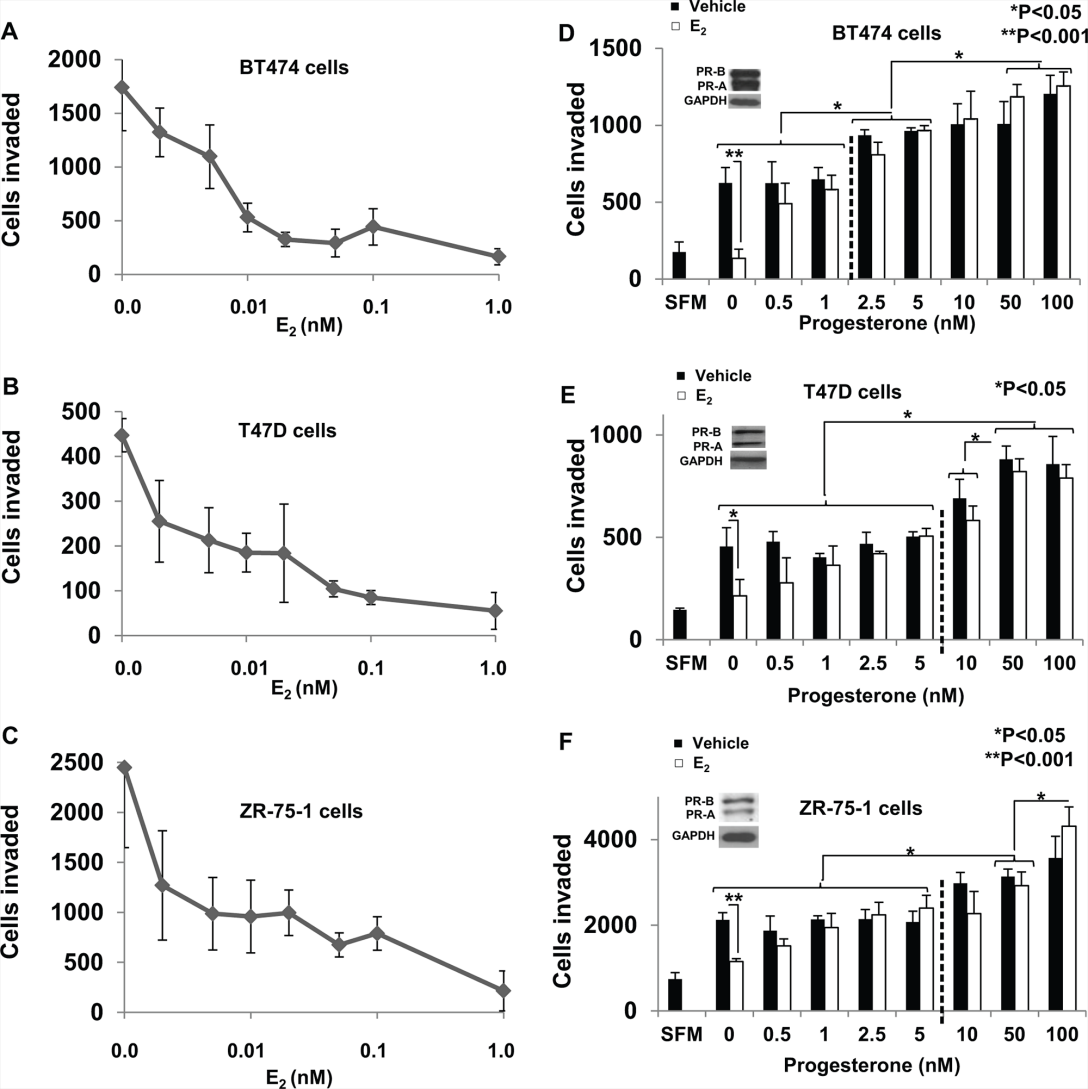
Dose response of regulation of breast cancer cell invasiveness by estrogen and progesterone In **panels A–C.** hormone depleted BT474 cells (*Panel A*), T47D cells (*Panel B*) and ZR-75-1 cells (*Panel C*) at 30% confluence were treated with vehicle or the indicated concentrations of E_2_ for 48 h. Cells were trypsinized and subjected to the matrigel transwell invasion assay with vehicle or the appropriate concentration of hormone present in the top and bottom chambers, as described under Materials and Methods. In the negative control, serum free media (SFM) was used instead of the FBS chemoattractant. Data points in the plots in *Panels A-C* represent values for invasiveness represented as average number of cells invaded with the background (SFM) values subtracted. In **panels D–F.** Hormone depleted BT474 cells (*Panel D*), T47D cells (*Panel E*) and ZR-75-1 cells (*Panel F*) at 30% confluence were treated with vehicle or E_2_ (1 nM) and the indicated concentrations of progesterone for 48 h. Cells were trypsinized and subjected to the matrigel transwell invasion assay with vehicle or the appropriate concentration of each hormone present in the top and bottom chambers, as described under Materials and Methods. In the negative control, serum free media (SFM) was used instead of the FBS chemoattractant. Insets show western blots of whole cell lysates from BT474 cells (*Panel D*), T47D cells (*Panel E*) and ZR-75-1 cells (*Panel F*) probed for PR and for GAPDH. In panels A–F, values for invasiveness are represented as average number of cells invaded from triplicate treatment sets and the error bars represent standard deviation. One-way ANOVA was performed on triplicate treatment sets and *P* values are indicated.

Estrogen (E_2_) is known to inhibit breast cancer cell invasiveness [47–51]. To relate the effect of E_2_ on invasiveness to physiological E_2_ levels, the E_2_ dose response for inhibition of invasiveness was determined in BT474, T47D and ZR-75-1 cells. E_2_ was able to inhibit invasiveness of the cells in the sub-nanomolar range with most of the inhibition occurring below 0.01 nM and virtually complete inhibition occurring at 0.1 nM in all three cell lines (Figure 1A, 1B, 1C). Thus the E_2_ dose that was required for substantial or virtually complete suppression of invasiveness in the three ER+ cell lines is at the low end of the literature consensus for both plasma and breast tissue levels of E_2_ in pre-menopausal (1.4nM–4.2nM) or post-menopausal (0.027 + 0.01 nM in plasma; 1.4nM + 0.7 in breast tisue) women [5, 6, 42, 43]. Invasiveness remained completely suppressed at higher concentrations of E_2_ (10nM and 20nM) (Supplemental Figure 1).

### Dose-dependent dual regulation of invasiveness by natural and synthetic progestins

Plasma levels of progesterone are known to change throughout a woman's menstrual cycle ranging from 0.6 nM to 4 nM in the follicular phase and upwards to greater than 50 nM in the luteal phase [44]. Furthermore the median plasma concentration of progesterone in post-menopausal women is 0.127 nM [45] with breast tissue concentrations an order of magnitude greater than in the plasma [7]. To examine the effects on progesterone in the context of estrogen signaling BT474, T47D and ZR- 75-1 cells were treated at varying concentrations (0 nM– 100 nM) of progesterone either alone or in the presence of a fixed concentration (1 nM) of E_2_ (Figure 1D, 1E, 1F). In all the three cell lines E_2_ alone inhibited invasiveness. However, progesterone at 0.5 nM at least partially rescued invasiveness from the effects of E_2_ and showed virtually complete rescue in all cases at a concentration of 1 nM. It may be noted that progesterone alone (in the absence of E_2_) did not influence invasion below a concentration of 2.5 nM – 5 nM but only rescued invasiveness from E_2_ regulation in the low concentration range (Figure 1D, 1E, 1F). At higher concentrations, progesterone progressively increased invasiveness of the cells independent of estrogen (Figure 1D, 1E, 1F). The hormones regulated invasiveness of the cells without affecting their migratory capacity (i.e., in the absence of matrigel in the transwells) (Supplemental Figure 2). Thus the data in Figure 1 (D–F) reveals two components of progesterone's effect on invasiveness *in vitro* in the three cell line models studied: (i) at low concentrations, progesterone rescues invasiveness from suppression by E_2_ and (ii) at higher concentrations, progesterone also induces invasiveness independent of E_2_.

**Figure 2:**
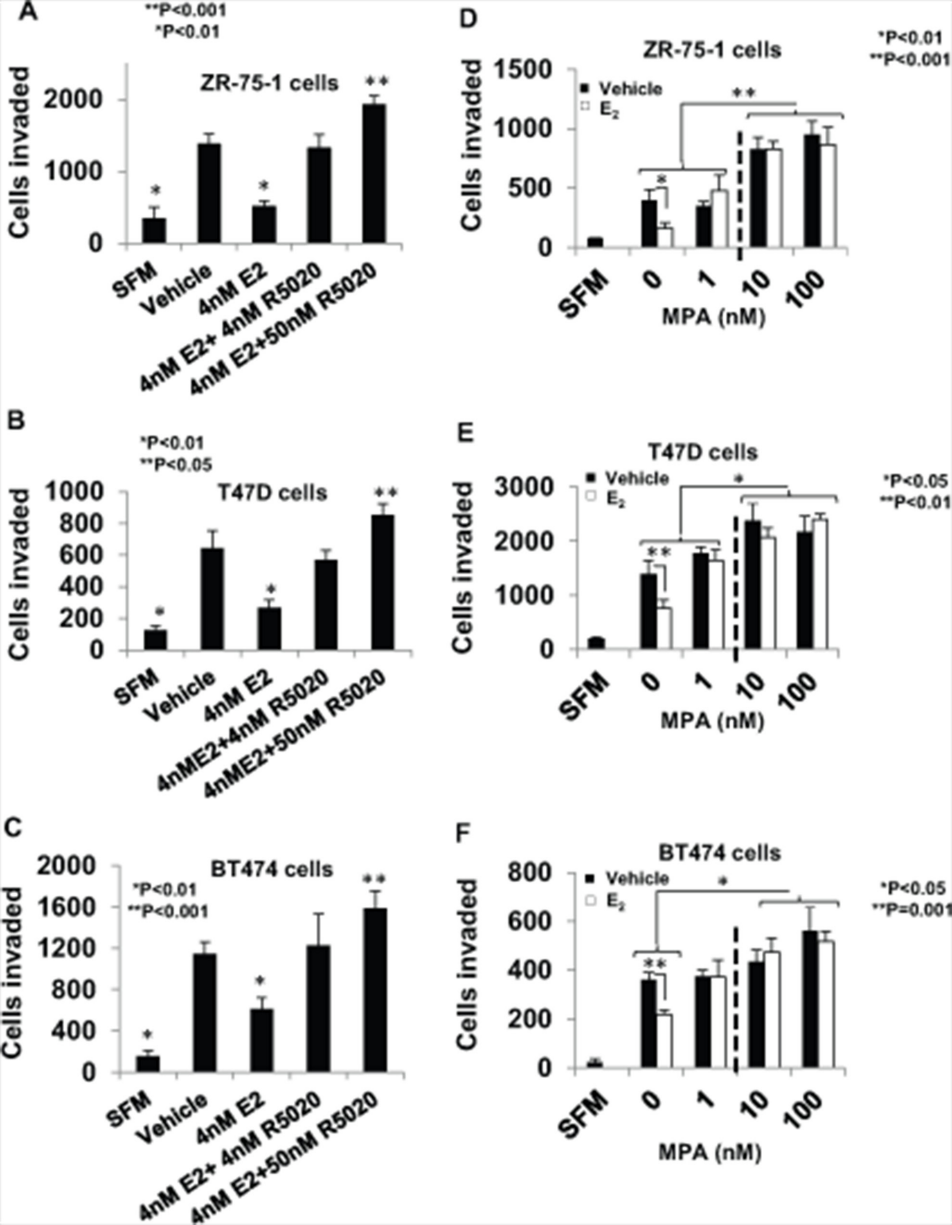
Regulation of breast cancer cell invasiveness by pre-menopausal concentrations of estrogen and progestin and dose-dependent effects of medroxyprogesterone acetate In **panels A–C.** hormone depleted ZR-75-1 cells *(Panel A)*, T47D cells *(Panel B)*, and BT474 cells *(Panel C)* at 30% confluence were treated with vehicle or E_2_ (4 nM), alone or in combination with R5020 (5 nM or 50 nM) for 48 h. Cells were trypsinized and subjected to the matrigel transwell invasion assay with vehicle or the appropriate concentration of E_2_ and/or R5020 present in the top and bottom chambers, as described under Materials and Methods. In the negative control, serum free media (SFM) was used instead of the FBS chemoattractant. In **panels D–F.** hormone-depleted ZR-75-1 cells *(Panel D)*, T47D cells *(Panel E)*, and BT474 cells *(Panel F)* cells at 30% confluence were treated with vehicle or the indicated concetrations of MPA either with or without 1nM E_2_ for 48 h. Cells were trypsinized and subjected to the matrigel invasion assay with vehicle or the appropriate concentration of E_2_ and/or MPA present in the top and bottom chambers, as described under Materials and Methods. In panels A–F, values for invasiveness are represented as average number of cells invaded from triplicate treatment sets and the error bars represent standard deviation. One-way ANOVA was performed on triplicate treatment sets and *P* values are indicated.

As noted above, during the luteal phase of the menstrual cycle, the E_2_ level is elevated to about 4 nM along with an increase in the progesterone levels from about 4 nM to about 50 nM. Therefore, we tested the effect of 4 nM and 50 nM R5020 (a more stable synthetic progestin), in the presence of 4 nM E_2_ on invasiveness of T47D, ZR-75-1 and BT474 cells (Figure 2A–2C). In all the three cell lines, suppression of invasiveness by E_2_ was completely prevented by both concentrations of R5020 and at 50 nM R5020, there was a further increase in invasiveness (Figure 2A–2C).

The dual effect of progesterone on invasiveness was recapitulated in all the three cell lines using the potent synthetic progestin medroxyprogesterone acetate (MPA). When MPA is used as a contraceptive (intramuscular route of administration) it has a mean plasma concentration of 2.58 nM [52] and has a 10–20 fold higher plasma concentration when administered orally during hormone replacement therapy [53]. At a concentration of 1 nM, MPA only reversed suppression of invasion by E_2_, but at higher concentrations (10 nM and 100 nM) MPA induced an increase in invasiveness well above the basal level whether or not E_2_ was present (Figure 2D–2F).

### The distinctive role of each PR isoform in the regulation of invasiveness by progestins

**Figure 3:**
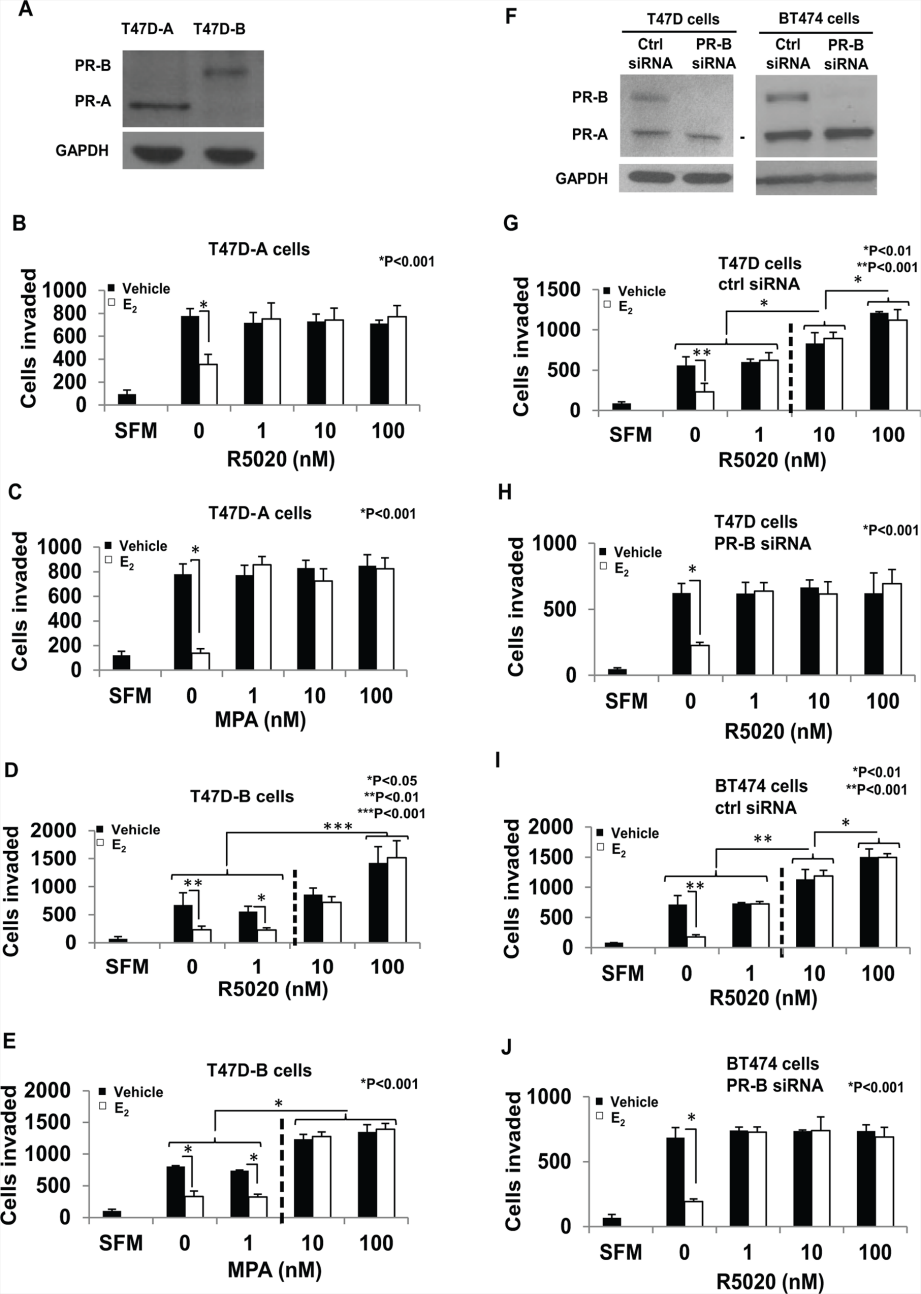
PR-A vs. PR-B mediated effects of progestins on invasiveness of breast cancer cells Panel A shows a western blot of cell lysates from T47D-A and T47D-B cells probed for PR or GAPDH (loading control); the position of the PR band is used to identify the PR isoform. In **panels A–E.** hormone-depleted T47D-A cells *(Panels B and C)* and T47D-B cells *(Panels D and E)* at 30% confluence were treated with vehicle or 1 nM E_2_ in combination with the indicated concentrations of R5020 (*Panels B and D*) or MPA (*Panels C and E*) for 48 h. Cells were trypsinized and subjected to the matrigel transwell invasion assay with vehicle or the appropriate concentrations of E_2_/R5020/MPA present in the top and bottom chambers, as described under Materials and Methods. In the negative control, serum free media (SFM) was used instead of the FBS chemoattractant. In **panels F–J.** hormone depleted cells were transfected with either siRNA directed against PR-B or non-targeted control siRNA and incubated for 48 h. In Panel F cell lysates from the transfected cells were prepared 96 h post-transfection and analyzed by western blot for PR or GAPDH (loading control); the PR isoforms are identified by their positions on the western blots. T47D cells transfected with control siRNA (*Panel G*) or PR-B targeted siRNA (*Panel H*) and also BT474 cells transfected with control siRNA (*Panel I*) or PR-B targeted siRNA (*Panel J*) were treated with vehicle or 1 nM E_2_ in combination with the indicated concentrations of R5020 for 48 h. Cells were trypsinized and subjected to the matrigel transwell invasion assay with vehicle or the appropriate concentrations of E_2_ and R5020 present in the top and bottom chambers, as described under Materials and Methods. In the negative control, serum free media (SFM) was used instead of the FBS chemoattractant. In panels B–E and G–J, values for invasiveness are represented as average number of cells invaded from triplicate treatment sets and the error bars represent standard deviation. One-way ANOVA was performed on triplicate treatment sets and *P* values are indicated.

The above observations led to the question of which PR-isoform(s) could mediate each of the two components of the regulation of invasiveness by progestins. To address this question, the effect of progestin dose on breast cancer cell invasiveness was tested in the absence or presence of 1nM E_2_ using recombinant T47D cells that exclusively express PR-A (T47D-A cells) or PR-B (T47D-B cells) (Figure 3A). The recombinant cells were a kind gift from Dr. Kathryn Horwitz who generated the cells as previously described [46]. Due to possible variance in absolute values of the number of cells invaded across experiments for a given cell line, we compared the invasive capacity of the T47D-A and T47D-B cells plated together under the same conditions at the same time. There was no difference in the invasive capacity of the two isogenic cell lines (Supplemental Figure 3). In T47D-A cells both R5020 and MPA only rescued invasiveness from E_2_ regulation at all concentrations tested (1nM, 10nM or 100 nM) but had no effect on invasiveness in the absence of E_2_ (Figure 3B and 3C). In contrast, in T47D-B cells, 1 nM of either R5020 or MPA was unable to rescue invasiveness from E_2_ regulation but at the higher concentrations (10 nM or 100 nM) they induced invasiveness well above the basal level and this was uninfluenced by the presence of E_2_ (Figure 3D and 3E).

As a complementary approach, we utilized siRNA knockdown of PR-B in parental T47D cells and in BT474 cells that express equal amounts of PR-A and PR-B. Both cell lines were transfected with siRNA directed against a target site in the unique 5′ segment of the PR-B mRNA or with control non-silencing siRNA using Lipofectamine. Knockdown of PR-B (western blots in Figure 3F) resulted in a loss of E_2-_independent induction of invasiveness at the higher concentrations (10 nM and 100 nM) of R5020 in both T47D cells and BT474 cells (Figure 3H and 3J) in contrast to the control siRNA transfected cells (Figure 3G and 3I). However the selective depletion of PR-B did not alter R5020′s ability to rescue invasiveness in the presence of E_2_, at all concentrations (1nM, 10nM, and 100nM) of R5020 (Figure 3G–3J). This result is consistent with those observed above using T47D-A cells.

The above results demonstrate that PR-A exclusively mediates the role of low dose progestins in opposing suppression of invasiveness by E_2_, whereas PR-B exclusively mediates E_2_-independent induction of invasiveness at high doses of progestins.

### Effect of RU486 on PR-A mediated induction of invasiveness by progestins

**Figure 4:**
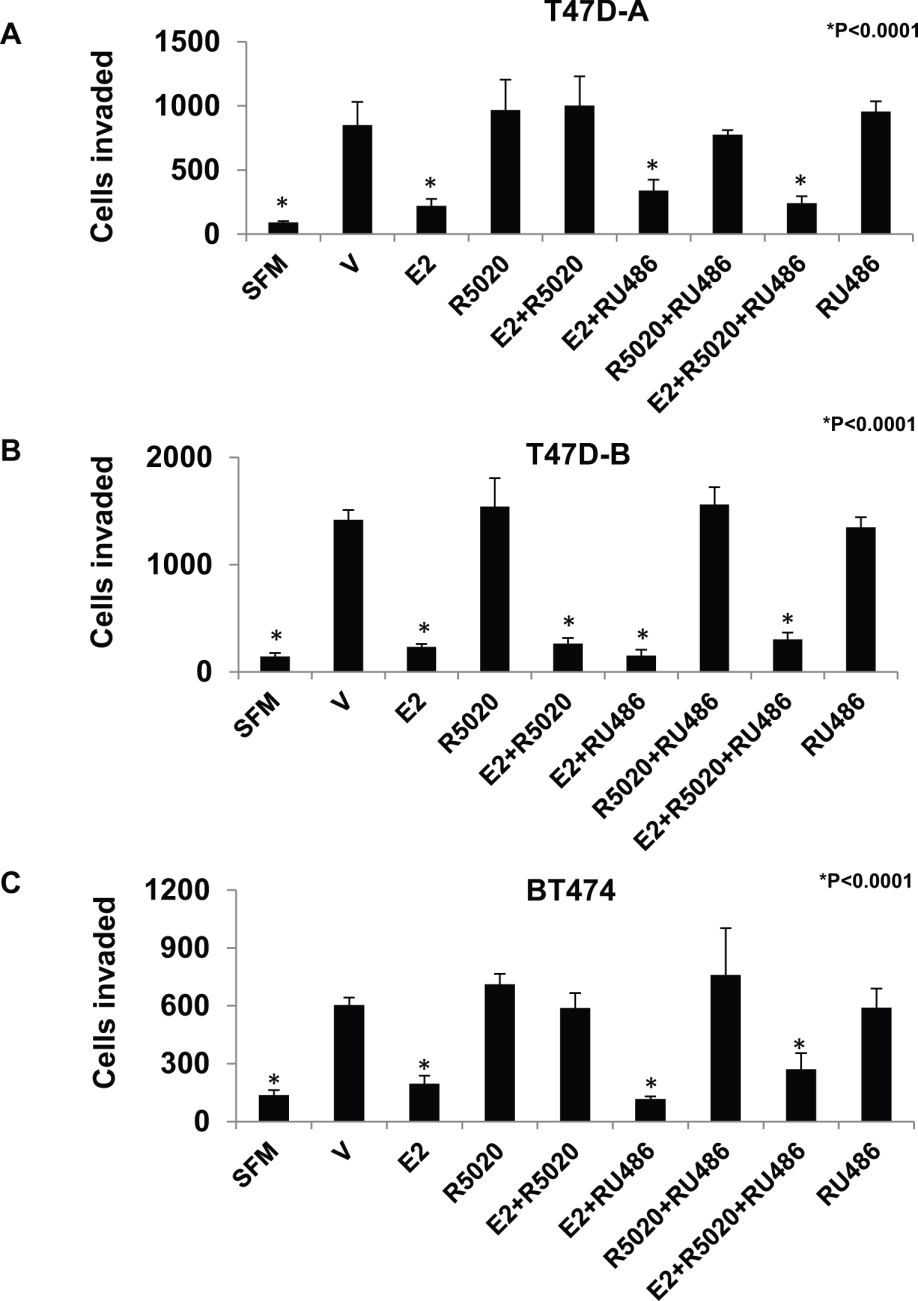
Effect of RU486 on regulation of breast cancer cell invasiveness by R5020 in relation to estrogen, PR-A and PR-B Hormone depleted T47D-A **Panel A.**, T47D-B **Panel B.** and BT474 **Panel C.** cells at 30% confluence were treated with vehicle or the indicated combinations of E_2_, R5020 and RU486, each at a concentration of 1nM for 48 h. Cells were trypsinized and subjected to the matrigel transwell invasion assay with vehicle or the appropriate concentration of E_2_, R5020 or RU486 present in the top and bottom chambers, as described under Materials and Methods. In the negative control, serum free media (SFM) was used instead of the FBS chemoattractant. Values are represented as average number of cells invaded from triplicate treatment sets and the error bars represent standard deviation. One-way ANOVA was performed on triplicate treatment sets and *P* values are indicated.

**Figure 5:**
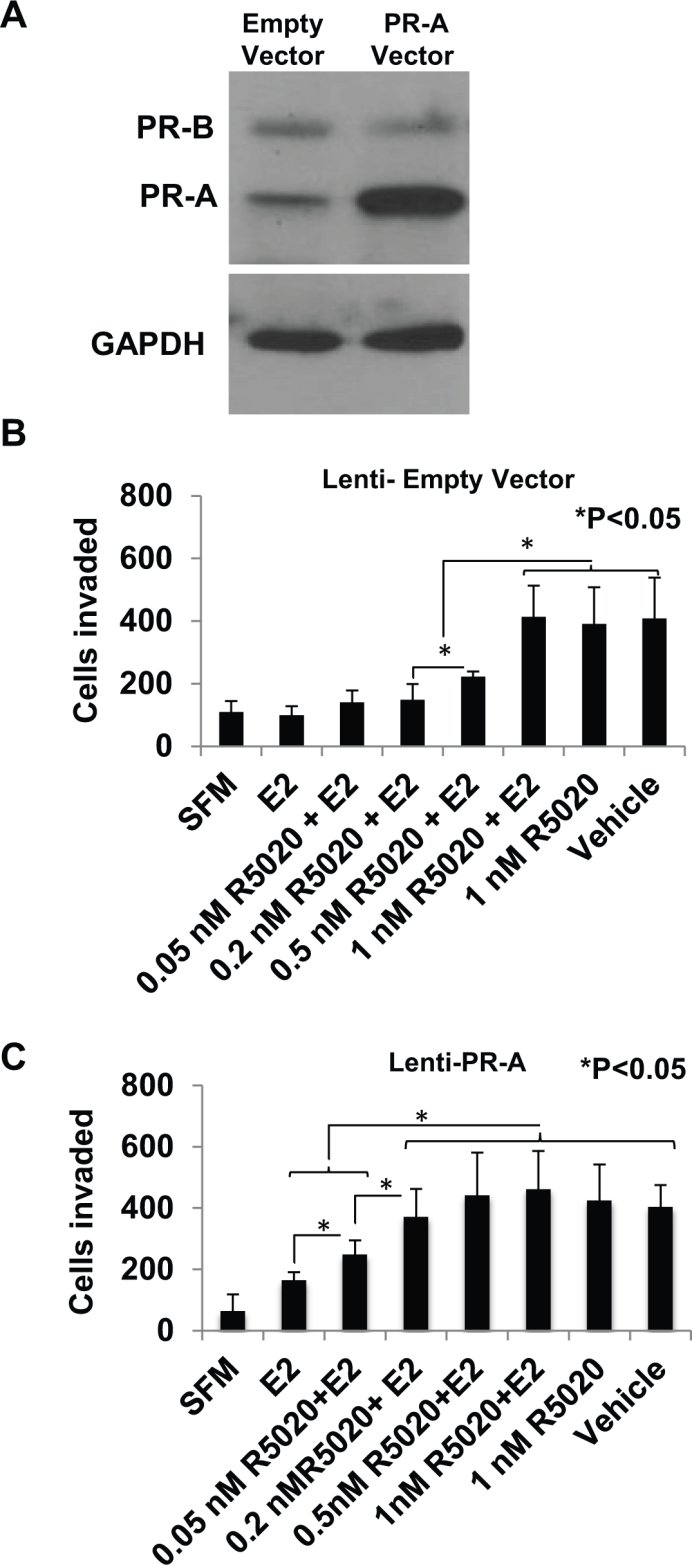
Effect of overexpressing of PR-A on the progestin dose response for rescue of invasiveness from estrogen regulation PR-A was ectopically overexpressed in hormone-depleted T47D cells by lentiviral transduction, as described under Materials and Methods. Whole cell lysates from cells transduced with either the PR-A expression vector or the control empty vector were probed for PR and for GAPDH **Panel A.** Cells transduced with the control empty vector **Panel B.** or PR-A expression vector **Panel C.** at 30% confluence were treated with vehicle or the indicated concentrations of R5020 in the absence or in the presence of E_2_ (1 nM) for 48 h. Cells were then trypsinized and subjected to the matrigel transwell invasion assay with vehicle or the appropriate concentration of E_2_ or R5020 present in the top and bottom chambers, as described under Materials and Methods. In the negative control, serum free media (SFM) was used instead of the FBS chemoattractant. Values are represented as average number of cells invaded from triplicate treatment sets and the error bars represent standard deviation. One-way ANOVA was performed on triplicate treatment sets and *P* values are indicated.

RU486 is a synthetic antagonist of progesterone that is PR isoform selective in specific target gene contexts. Therefore it was of interest to test the effect of RU486 on the PR-A dependent actions of progesterone on breast cancer cell invasiveness. The cell lines T47D-A, T47D-B and BT474 were treated with either E_2_, the progestin R5020, or the anti-progestin RU486, each at a concentration of 1 nM in the various combinations indicated in Figure 4. In T47D-A cells, RU486 disrupted the ability of R5020 to rescue invasiveness from E_2_ suppression but did not have any effect by itself on invasiveness, in either the presence or absence of E_2_ (Figure 4A). On the other hand, in T47D-B cells, RU486 had no effect on invasiveness under any of the conditions tested when each of the ligands was used at a concentration of 1 nM (Figure 4B). In BT474 cells, which express equal amounts of both PR isoforms, the effect of RU486 was similar to that observed in the T47D-A cells, demonstrating that agonist or antagonist actions that modulate the effect of PR-A on invasiveness are functionally independent of PR-B expression (Figure 5C).

### Hypersensitization of PR-A to progestin through overexpression of the receptor

As noted above, PR-A is frequently overexpressed in invasive clinical breast tumors. It was therefore of interest to examine the possibility that overexpression of PR-A in the tumor cells may sensitize PR-A mediated regulation of invasiveness to post-menopausal breast tissue levels of progesterone.

T47D cells express comparable amounts of PR-A and PR-B protein as observed on a western blot probed with an antibody against a common carboxyl-terminal peptide of the two receptor isoforms (Figure 5A). Lentiviral transduction of a PR-A expression plasmid increased the level of PR-A by approximately 3.7-fold, without altering the expression of PR-B (Figure 5A). The R5020 dose-dependence for rescue of invasiveness from E_2_ regulation was compared between the PR-A overexpressing cells and the control cells transduced with the empty vector. Overexpression of PR-A clearly conferred hypersensitivity to R5020 as the progestin partially rescued invasiveness even at a concentration of 0.05 nM and fully rescued invasiveness at a concentration of 0.2 nM in the PR-A overexpressing cells (Figure 5C); in comparison, in the control cells a concentration of 0.5 nM – 1.0 nM R5020 was required to observe similar effects (Figure 5B).

### PR isoform A-dependent regulation of E_2_ target genes by progestin and their functional role

**Figure 6:**
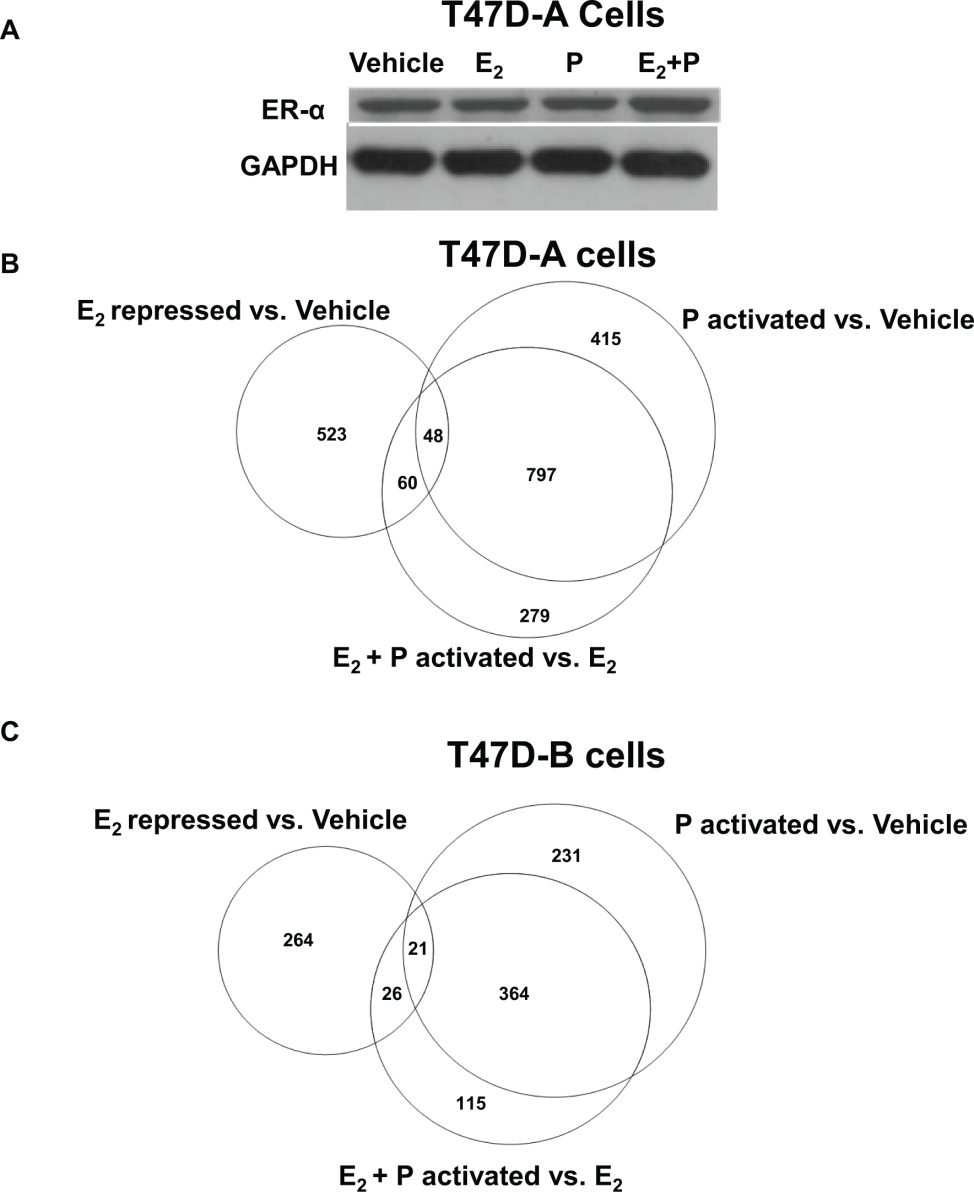
Effect of low dose progestin on the gene repression profile of estrogen in relation to PR-A and PR-B Hormone depleted T47D-A or T47D-B cells at 30% confluence were treated with vehicle, 1 nM E_2_, 1 nM R5020 (P) or 1nM E_2_ plus1nM R5020 (E_2_ + P) for 48 h. In **Panel A.** whole cell lysates from the treated T47D-A cells were probed by western blot for ERα and for GAPDH. In parallel, total RNA was extracted from the treated T47D-A and T47D-B cells and subjected to mRNA expression profiling as described under Materials and Methods. The mRNA profiling data is represented in the Venn diagrams in **Panel B.** (for T47DA cells) and in **Panel C.** (for T47D-B cells).*Panels B* and *C* show comparisons among the gene set repressed by E_2_ (E_2_ repressed vs. Vehicle), the gene set activated by R5020 in the absence of E_2_ (P activated vs. Vehicle) and the gene set activated by R5020 in the presence of E_2_ (E_2_ + P activated vs. E_2_). The data represents results from experimental triplicates.

E_2_ acts through its receptor ER to repress expression of genes known to be involved in breast tumor invasion, EMT, and metastasis [49, 50, 54–56]. The ability of progestins to oppose E_2_ regulation of invasiveness did not involve a decrease in ER expression as evident from a western blot of T47D-A cells treated with R5020 (Figure 6A).

**Figure 7:**
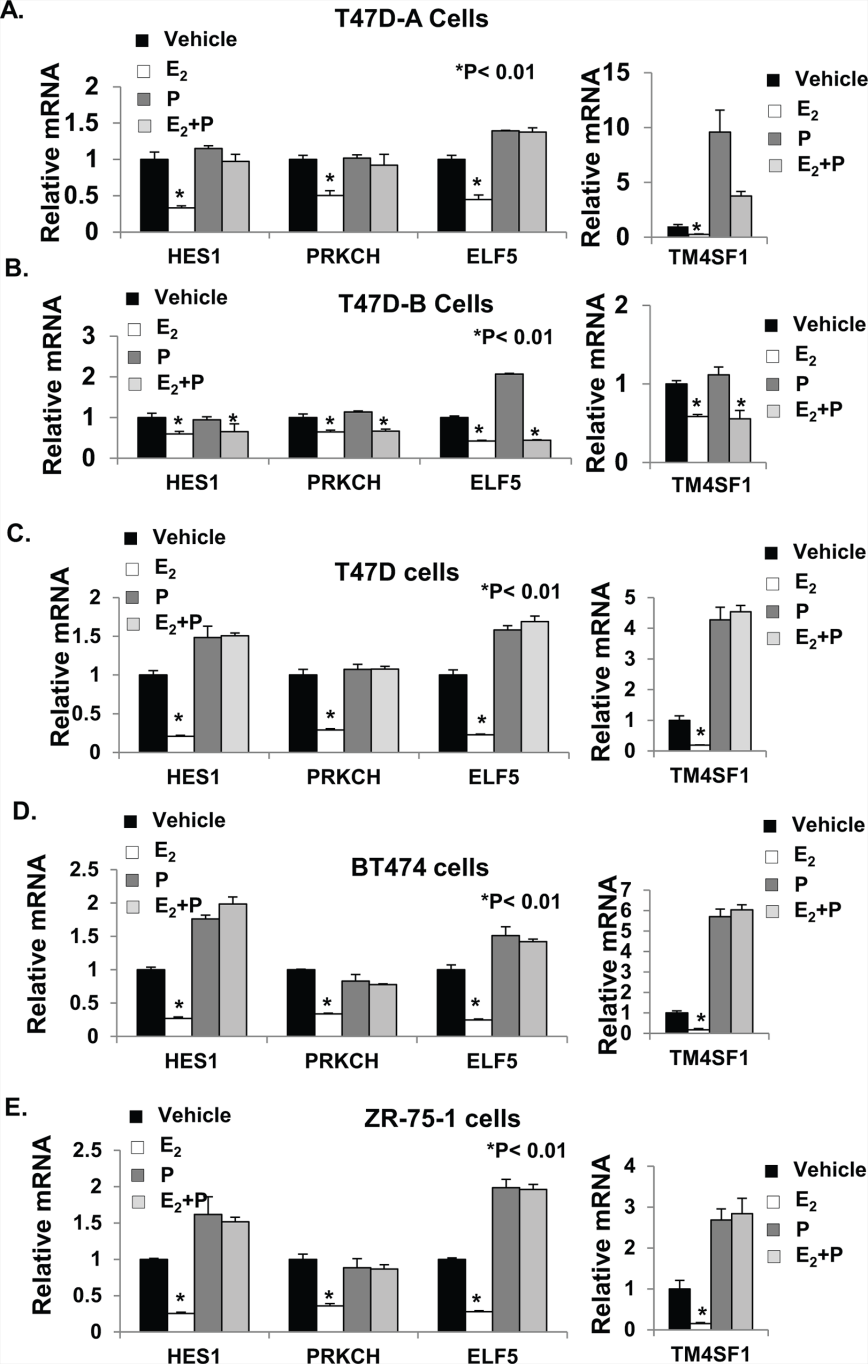
Validation of gene expression profiling The total RNA samples used for mRNA expression profiling in Figure 6 were used for validation of the mRNA profiling data for selected genes in T47D-A cells **Panel A.** and T47D-B cells **Panel B.** Validation of estrogen and progestin regulation of these genes in the PR-A+ cells was also extended to T47D (parental) cells **Panel C.** BT474 cells **Panel D.** and ZR-75-1 cells **Panel E.** RNA purified from the treated cells was reverse transcribed and the cDNA was analyzed by real-time PCR using TaqMan Probes, as described under Materials and Methods. Relative mRNA levels were measured in the samples for HES1, PRKCH, ELF5, and TM4SF1 genes. All C_T_ Values were normalized to GAPDH and represented as fold change in comparison to vehicle treated controls. The mRNA values are the average (+/− standard deviation) from triplicate assays performed for each one of the triplicate treatment sets.

Next, we undertook to examine PR isoform-specific effects on transcriptional signaling by E_2_ using T47D-A or T47D-B cells. A concentration of 1 nM R5020 was chosen because at this concentration the progestin completely rescued invasiveness from E_2_ regulation (through PR-A) but did not exert E_2_-independent effects on invasiveness (through PR-B) (please refer back to Figure 3B and 3D). The cells were treated with vehicle, 1 nM E_2_, 1 nM R5020 or 1 nM R5020 + 1 nM E_2_ for an extended duration of 48 hours to examine expression of both direct and indirect target genes of the hormones. mRNA expression profiles were examined by DNA microarray analysis using the Illumina platform and an arbitrary cut off value of 1.5-fold was applied to identify patterns of changes in mRNA expression. In T47D-A cells, among 631 genes that were repressed by E_2_ (Supplemental Table 1) (Figure 6B), R5020 opposed the repression of 108 genes (Supplemental Table 2) (Figure 6B) including 48 genes that were activated by R5020, independent of E_2_ (Supplemental Table 3) (Figure 6B). In T47D-B cells, among 311 genes that were repressed by E_2_ (Supplemental Table 4) (Figure 6C), R5020 opposed the repression of 47 genes (Supplemental Table 5) (Figure 6C) including 21 genes that were activated by R5020, independent of E_2_ (Supplemental Table 6) (Figure 6C). Inspection of these gene lists revealed that of the 108 E_2_ repressed genes whose expression was rescued by R5020 in T47D-A cells, only 9 genes were also rescued by R5020 in T47D-B cells. The E_2_ repressed genes that were activated by progesterone alone were also cell-type specific, with only 8 exceptions. Thus, repression of 99 genes by E_2_ was opposed by R5020 in an exclusively PR isoform A-dependent manner. We next searched the literature to identify all the genes in this group that had suggested or established roles in breast tumor biology. A total of 19 genes were clearly known to be associated with breast tumor biology and they predominantly supported breast tumor progression, including invasiveness and metastasis (Supplemental Table 7). The DNA microarray data is validated for 4 representative genes (HES1, PRKCH, ELF5 and TM4SF1) by quantitative real time RT-PCR in Figure 7A and 7B using T47D-A and T47D-B cells. We also confirmed that these four genes were regulated in T47D (parental), BT474, and ZR-75-1 cells in the same pattern as that observed in T47D-A cells (Figure 7C–7E).

**Figure 8:**
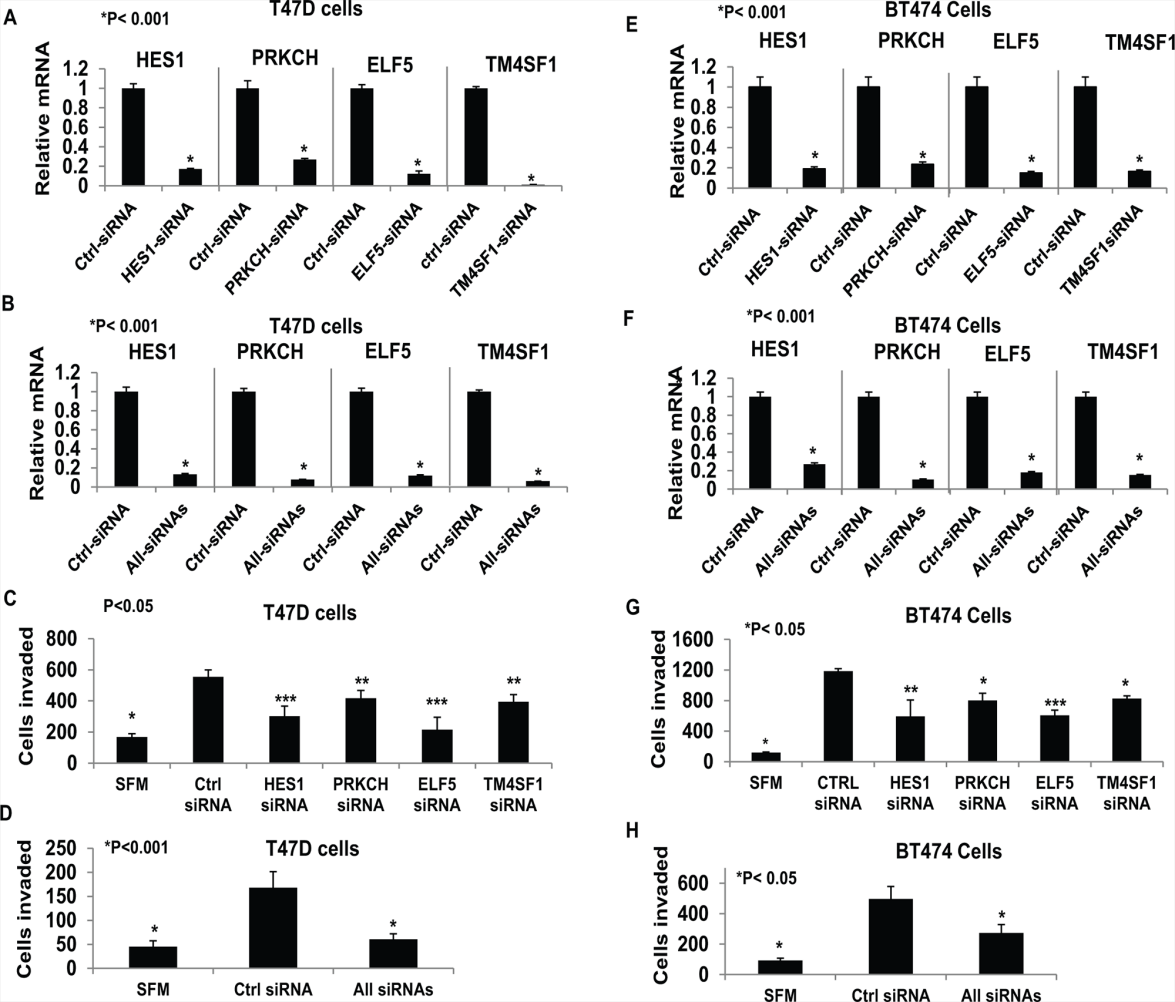
Functional testing of selected tumor progression genes Hormone-depleted T47D cells **Panels A–D.** and BT474 **Panels E–H.** were transfected with control siRNA, TM4SF1 siRNA, HES1 siRNA, ELF5 siRNA and PRKCH siRNA independently (*Panel A, C, E, and G)* or all four targeted siRNAs in combination (*Panels B, D, F, H*). After 72 hours cell were subjected to the transwell matrigel invasion assay (*Panels C, D, G, and H*) as described under Materials and Methods. In the negative controls, serum free medium (SFM) was used instead of the FBS chemoattractant. Values are represented as average number of cells invaded from triplicate treatment sets and the error bars represent standard deviation. One way ANOVA was performed on triplicate treatment sets and *P* values are indicated.

The four genes (HES1, PRKCH, ELF5 and TM4SF1) validated above have all been associated with cancer progression. To directly test whether regulation of these genes by E_2_ mediated the hormonal effects of E_2_ on invasiveness in ER+ breast cancer cells, we used a loss-of-function approach. T47D and BT474 cells were transfected with siRNAs against the four genes either individually (Figure 8A and 8E) or together (Figure 8B and 8F); in all cases, the siRNAs effectively knocked down the genes, as observed by their mRNA levels compared to the cells transfected with control non-targeted siRNA (Figure 8A, 8B, 8E and 8F). For the protein products of the genes for which high quality antibodies were available (i.e., ELF5 and HES1), the knockdown was also confirmed by western blot (Supplemental Figure 4). Individually knocking down the genes decreased invasiveness of the cells to different degrees (Figure 8C and 8G) and the combined knockdown completely suppressed invasiveness (Figure 8D and 8H). The results indicate the functional relevance of genes whose regulation by E_2_ was found in this study to be opposed by low dose progesterone acting through PR-A. Clearly the subset of E_2_ repressed genes that are counter-regulated by progesterone/PR-A include genes that mediate hormonal regulation of invasiveness in breast cancer cells.

**Figure 9:**
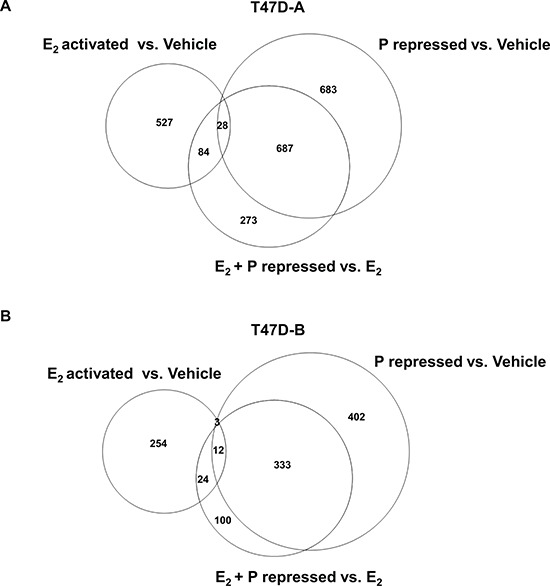
Effect of low dose progestin on the gene activation profile of estrogen in relation to PR-A and PR-B Hormone depleted T47D-A or T47D-B cells at 30% confluence were treated with vehicle, 1 nM E_2_, 1 nM R5020 (P) or 1nM E_2_ plus1nM R5020 (E_2_ + P) for 48 h. Total RNA was extracted from the treated T47D-A and T47D-B cells and subjected to mRNA expression profiling as described under Materials and Methods. The mRNA profiling data is represented in the Venn diagrams in **Panel A.** (for T47DA cells) and in **Panel B.** (for T47D-B cells). *Panels A* and *B* show comparisons among the gene set activated by E_2_ (E_2_ activated vs. Vehicle), the gene set repressed by R5020 in the absence of E_2_ (P repressed vs. Vehicle) and the gene set repressed by R5020 in the presence of E_2_ (E_2_ + P repressed vs. E_2_). The data represents results from experimental triplicates.

A similar analysis was then conducted for E_2_ activated genes in T47D-A (Figure 9A and Supplemental Tables 8–10) and T47D-B (Figure 9B and Supplemental Tables 11–13) cells. We found that activation of 112 genes by E_2_ was opposed by R5020 in an exclusively PR-A isoform dependent manner. Within this group, the small number of genes with better known functions in breast tumor biology tended to support growth and inhibit invasiveness.

## DISCUSSION

The results of this study reveal that the positive effect of progestins on invasiveness of ER+ breast cancer cells has two components: 1. rescue of invasiveness from estrogen repression at relatively low progestin concentrations that is mediated exclusively by PR isoform A and 2. estrogen-independent induction of invasiveness at high progestin concentrations that is mediated exclusively by PR isoform B. Moreover, PR-A was sensitized to even lower levels of progestin when this receptor isoform was overexpressed relative to PR-B. Similar to the observations here on PR isoforms, other steroid receptors are also known to induce distinct genotropic and phenotypic effects at different hormone doses as well as hypersensitization to hormone by a few fold overexpression of the receptor [41, 57].

The relevance of the above findings to the physiological hormone status prior to and after menopause is apparent. The estrogen dose that was required for substantial or virtually complete suppression of invasiveness in ER+ cells is well under the plasma levels of estrogen in pre-menopausal women. It is also within the range of plasma and breast tissue levels of estrogen in post-menopausal women. The full effect of PR-A on the invasiveness of the various breast cancer cell lines occurred at < 1 nM progesterone and the dose requirement was reduced to < 0.2 nM when the expression level of PR-A was elevated. Thus, dysregulated PR-A has the potential to rescue invasiveness of breast cancer cells from estrogen regulation in response to post-menopausal plasma/breast tissue progesterone levels. This is in contrast to PR-B, which only induced invasiveness progressively with progesterone dose in the range of 5 nM to 50 nM. Thus, isoform A of PR plays the predominant hormone-dependent role in increasing invasiveness of ER+ breast cancer cells at progesterone concentrations that include the entire range of follicular phase, luteal phase and post-menopausal hormone levels, particularly when the cells overexpress PR-A. The findings on the role of PR isoforms also extend to plasma progestin levels associated with the use of MPA, either in contraception or in hormone replacement therapy. Therefore in luminal breast cancer, prior to diagnosis or after cessation of treatment, PR-A may have a greater mechanistic role in promoting invasiveness than PR-B.

The unique ability of only isoform A of PR to oppose regulation of invasiveness by estrogen at low progesterone concentrations is clearly reflected in the differential abilities of PR-A and PR-B to mediate cross-talk between progesterone and estrogen with respect to patterns of gene regulation. Gene expression analysis using isogenic recombinant (T47D) cells that exclusively expressed either the A or the B isoform of PR revealed that the cross-talk between estrogen and low dose progesterone affected the expression of estrogen target genes with diverse functions. However, among these genes, the subsets that were regulated by progesterone through PR-A vs. PR-B were largely non-overlapping. The genes whose regulation by estrogen was opposed by progesterone in an exclusively PR-A dependent manner included both estrogen-activated and estrogen-repressed genes. The estrogen-repressed genes were more noteworthy in the context of this study as they included genes with established roles in progression of breast cancer. Moreover, we demonstrated that selected genes from this subset (HES1, PRKCH, ELF5 and TM4SF1) did support invasiveness in ER+ breast cancer cells.

In response to the binding of progesterone, several mechanisms could conceivably enable PR-A to oppose estrogen's action on a subset of estrogen target genes. The ligand-dependent activity of PR-A did not result in any change in ER expression. Rather, the exact mechanism of PR-A isoform dependent cross-talk between progesterone and estrogen signaling could depend on the target gene context. For example, (i) PR-A could compete with ER to bind to tethering proteins at repressive sites in the chromatin, either simply blocking repression by estrogen/ER or activating the target gene; (ii) PR-A could bind at chromatin sites that are different from the repressive sites of ER binding and compete with ER for interaction with the pre-initiation complex of the target genes; (iii) PR-A could indirectly oppose gene regulation by estrogen by regulating transcription of other regulatory proteins or microRNAs. The amino-terminal truncation in PR-A could expose protein binding motifs that are unexposed in PR-B enabling unique or higher affinity interactions of agonist bound PR-A with other regulatory proteins in the chromatin. Similar chromatin interactions of PR-B may therefore require higher doses of progestins. More extensive studies including ChIP-seq analyses should help to establish specific mechanisms by which PR-A may de- regulate estrogen target genes.

In mice, selective ablation of PR-B revealed that PR-B was not required for the normal physiology ofthe uterus or the ovary but was necessary for pregnancy-associated mammary gland morphogenesis [58]. That study demonstrated that the ability of progesterone to suppress estrogen-induced endometrial proliferation was due to PR-A. In contrast, when PR-A was selectively ablated, progesterone not only failed to inhibit estrogen-induced cell proliferation in the endometrium but actually further increased proliferation of the uterine epithelium, an effect mediated by PR-B [59]. Therefore, given the necessary role of PR-A in endometrial physiology, selectively disrupting its actions in breast cancer cells vs. endometrial tissue will require a better understanding of tissue-specific molecular pathways by which PR-A opposes estrogen signaling in breast cancer. Identifying and narrowly targeting a critical cross-talk pathway between PR-A and ER may enable suppression of tumor progression without disrupting the protective role of PR-A in the endometrium or the adverse effects of a broader PR antagonist. Such an intervention may also be useful in combination hormone replacement therapy. A molecular signature of hyperactive PR-A may also more effectively predict tumor progression.

## MATERIALS AND METHODS

### Chemicals and reagents

Dulbecco's modified Eagles medium (DMEM) and phenol red-free DMEM, glutamine, penicillin, streptomycin, Fetal Bovine Serum (FBS), charcoal stripped FBS and TaqMan probes were purchased (Life Technologies, Carlsbad, CA). 17β-estradiol (E_2_), R5020, RU486, progesterone and medroxyprogesterone acetate (MPA) were purchased from Sigma Aldrich (Saint Louis, MO). Growth factor reduced matrigel (Cat# 356231) and calcein AM fluorescent dye (Cat# 354216) were purchased from BD Biosciences (San Jose, CA). PR-B directed siRNAs [60, 61] and control non-silencing siRNA (Cat# SIC001) were ordered from Sigma Aldrich (St. Louis, MO). siRNAs targeting TM4SF1 (Cat# S8367), HES1 (Cat# S6920), PRKCH (CAT#S1107), and ELF5 (CAT# S4629) were purchase from Life Technologies (Carlsbad, CA).

### Cell culture and treatment

BT474, T47D and ZR-75-1 breast cancer cells (American Type Culture Collection) were cultured in DMEM supplemented with FBS (10%) penicillin (100 unit/ml) streptomycin (100ug/ml) and L-glutamine (2 mM). T47D-A and T47D-B cells were a generous gift from Dr. Katherine Horowitz (University of Colorado, Denver, CO) and were cultured as previously described [16]. The cell lines were all cultured at 37°C with 5% CO_2_. Before hormone treatment, cells were plated in 6-well plates at 30% confluence in phenol red-free media supplemented with charcoal-stripped FBS and incubated at 37°C with 5% CO_2_ for 48 h. Cells were then treated with vehicle, progesterone, MPA, R5020, RU486 and E_2_ alone or in various combinations at concentrations as indicated for each individual experiment for a duration of 48 h. The cells were then harvested for mRNA analysis, western blot analysis or cell invasion assays.

### Western blot analysis

Cells were lysed using RIPA Buffer (150 mM NaCl, 1% NP-40, 0.5% sodium deoxycholate, 1% SDS, and 50 mM Tris pH 8.0) containing protease inhibitor cocktail (Pierce Biotechnology, Rockford IL). The lysates were chilled on ice and agitated by vortex every ten minutes for one hour. Total protein concentration was measured by Bradford assay (Bio-Rad laboratories, Hercules, CA). A total amount of 10–40 μg protein per sample was resolved by electrophoresis on a 8% SDS-polyacrylamide gel and transferred to a PVDF membrane (Millipore Corporation, Bedford MA). Membranes were probed with primary polyclonal rabbit anti-PR antibody (sc-539, Santa Cruz biotechnologies, CA), polyclonal rabbit anti-ERα antibody (sc-543, Santa Cruz Biotechnologies, CA), mouse monoclonal anti-GAPDH antibody (sc-4472, Santa Cruz Biotechnologies, CA), rabbit polyclonal anti-HES1 (sc-25392, Santa Cruz Biotechnologies, CA) or mouse monoclonal anti-ELF-5(sc-376737, Santa Cruz Biotechnologies, CA). The blots were then probed with appropriate horseradish peroxidase conjugated secondary antibody (Vector Laboratories, MD). The protein bands were visualized using enhanced chemiluminescence reagent Hyglo Quick spray (Denville Scientific, South Plainfield, NJ) per the manufacturer's suggested protocol. Relative protein expression was determined by ImageJ (National Institutes of Health, USA).

### RNA isolation, reverse transcription PCR and real time PCR

Total RNA was isolated using the RNeasy mini kit (Qiagen, MD). Reverse transcription PCR reactions were performed using high capacity complementary DNA archive kit (Life Technologies Corporation, Carlsbad, CA) according to manufacturer's protocol. cDNA was measured by quantitative real time PCR using the StepOne Plus Real time PCR system (Life technologies Corporation, Carlsbad, CA). All mRNA measurements were performed in biological triplicates, and all C_T_ values were normalized to intra-sample GAPDH. mRNA values were represented as fold difference, which is calculated using the formula = 2^−ΔΔC^_T_, where ΔΔC_T_ = ΔC_T_ sample − ΔC_T_ calibrator (ΔC_T_ = C_T_ of gene of interest- C_T_ of GAPDH).

### Boyden chamber transwell invasion assay

Cells (1 × 10^5^) were re-suspended in the appropriate culture media devoid of serum and phenol red and added to the top chamber of the flouroblok inserts (Cat# 351152, 8 μM pore membrane: BD biosciences, Bedford, MA) coated with growth factor reduced matrigel (0.2 mg/ml). The chemoattractant comprised phenol red-free media supplemented with FBS (20%). The appropriate hormone treatment was included in both the top and bottom chambers. Each treatment was replicated in three wells and the entire experiment was replicated at least three times. Cells were allowed to invade for 24 h at 37°C with 5% CO_2_. Cells that invaded to the bottom surface were stained with calcein AM (4ug/ml) in serum free media in the dark for 1 h at 37°C with 5% CO_2_. Images were captured in an identical manner from each well in 5 non-overlapping fields (the middle of the well and surrounding fields) using a 4x objective. Images were analyzed using ImageJ software (National Institutes of Health, USA) and the number of cells invaded was quantified by brightness and pixel size.

### Migration Assay

Pre-treatment and preparation of cells and the experimental protocol were identical to those described above for the Boyden chamber transwell invasion assay with the exception that the transwells were devoid of matrigel.

### Lentiviral transduction

293FT cells were used to generate lentiviral particles by transfection using lipofectamine 2000 (Life Technologies Corporation, Carlsbad, CA). Packaging plasmids pMD2G, PMDLg/RRE, and pRSV/Rev were cotransfected with pCDH PR-A expression plasmid, or pCDH empty vector plasmid. Lentivirus containing supernatant was harvested at 48 h and 72 h after transfection. T47D cells were plated in phenol red-free DMEM supplemented with heat-inactivated charcoal-stripped FBS (10%) and 2 mM L-Glutamine two days before infection. For infection, T47D cells were transduced with either pCDH empty vector lentivirus or pCDH PR-A lentivirus with polybrene (8 μg/ml) for 5 h. A second transduction was performed similarly for another 5 h. The cells were then incubated in phenol red-free DMEM supplemented with charcoal-stripped serum (10%) and L-Glutamine (2 mM) for 48 h. Following infection, cells were harvested for western blots and cell invasion assays as described previously.

### siRNA Transfection

Cells were plated to 30% confluence without antibiotic in phenol-red free DMEM medium supplemented with 10%charcoal-stripped FBS. 24 hours later cells were transfected with siRNA directed against specific gene targets or non-silencing siRNA using lipofectamine (Life Technologies, Carlsbad, CA) according to the manufacturer's protocol.

### mRNA expression profiling

T47D-A and T47D-B cells were depleted of hormone for 48 h as described above. Cells were then either treated with vehicle, 1nM E_2_, 1nM R5020, or 1nM E_2_+ 1nM R5020 for 48 h. Total RNA was isolated using the RNeasy mini kit (Qiagen, MD). Sample identities were randomized for blinded analysis. The samples were analyzed at the Wayne State University School of Medicine Applied Genomics Center (AGTC) using the HumanHT-12 v4 Expression BeadChip with the Illumina HiScan System (Illumina, San Diego, CA). A total of 47,000 probes were used to analyze the transcriptome expression for each treatment group. Data was analyzed using Partek V6.6 software (St. Louis, MO), and processed using genome Studio (Illumina, San Diego, CA). Expression values were normalized using quantile-normalization, with background subtraction. Log transformation to the base of 2, followed by one way ANOVA was used to determine error. The differentially expressed genes were identified by comparing E_2_ treatment with vehicle treatment, R5020 treatment with vehicle treatment and E_2_ treatment with E_2_+ R5020 treatment (repressed or activated with a fold difference of 1.5 and a *p* value < 0.05). Genes that had activated expression in E_2_+ R5020 treatment but were repressed by E_2_ treatment were identified. Genes that were activated in E_2_ treatment but repressed by E_2_+ R5020 were also identified. Gene ontology analysis was performed by literature mining by searching the MEDLINE database (National institutes of Health, USA) with a query of “name of gene” followed by the term “AND Cancer” or “ AND Breast Cancer”. All articles under the specified query were examined to determine gene function in breast cancer. Validation of Microarray Data was performed by real-time RT-PCR as described above using TaqMan probes.

### Statistical analysis

Experimental values were presented as mean +/− standard deviation using triplicate treatment sets. The statistical difference between values was determined by using one way ANOVA followed by post hoc paired *t*-test. The significant *P* values are noted in the figures. Concordant results were obtained from at least three repetitions of the experiments conducted on different days.

## SUPPLEMENTARY MATERIALS FIGURES AND TABLE




























