# Supplementary material for: Role of the short isoform of the progesterone receptor in breast cancer cell invasiveness at estrogen and progesterone levels in the pre- and post-menopausal ranges
Source: Oncotarget. 2015 Aug 24;6(32):33146–64. doi: 10.18632/oncotarget.5082 (PMC4741755; doi:10.18632/oncotarget.5082)
Supplement: Supplementary file 1 [file oncotarget-06-33146-s001.pdf]

SUPPLEMENTARY FIGURES AND TABLE

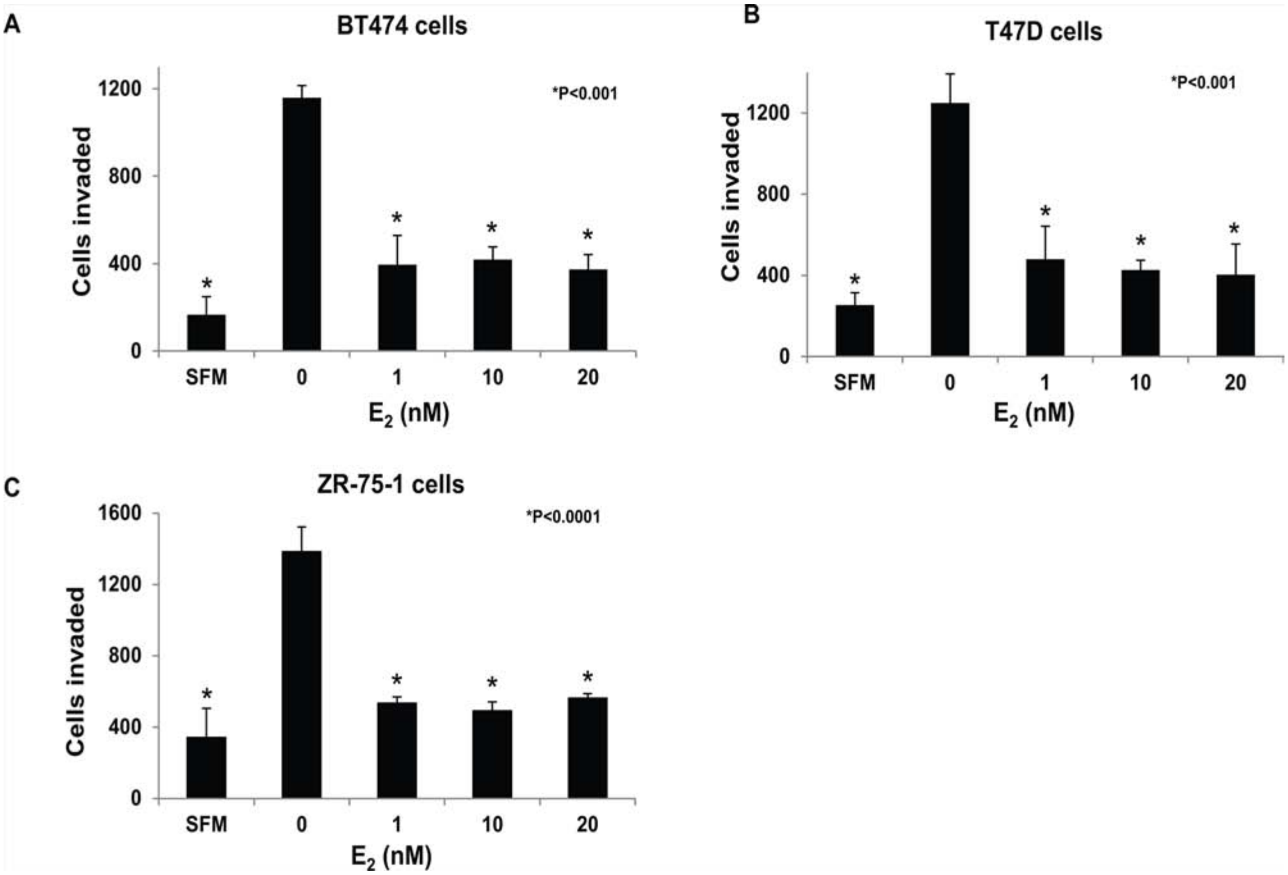

Supplementary Figure S1: Inhibition of invasiveness by estrogen at different concentrations. Hormone depleted BT474cells **Panel A.**, T47D cells **Panel B.** and ZR-75–1 cells **Panel C.** at 30% confluence were treated with vehicle or the indicated concentrations of E_2 for 48 h. Cells were trypsinized and subjected to the matrigel transwell invasion assay with vehicle or the appropriate concentration of hormone present in the top and bottom chambers, as described under Materials and Methods. In the negative control, serum free media (SFM) was used instead of the FBS chemoattractant. Values are represented as average number of cells invaded from experimental triplicates and the error bars represent standard deviation. *P* Values are indicated.

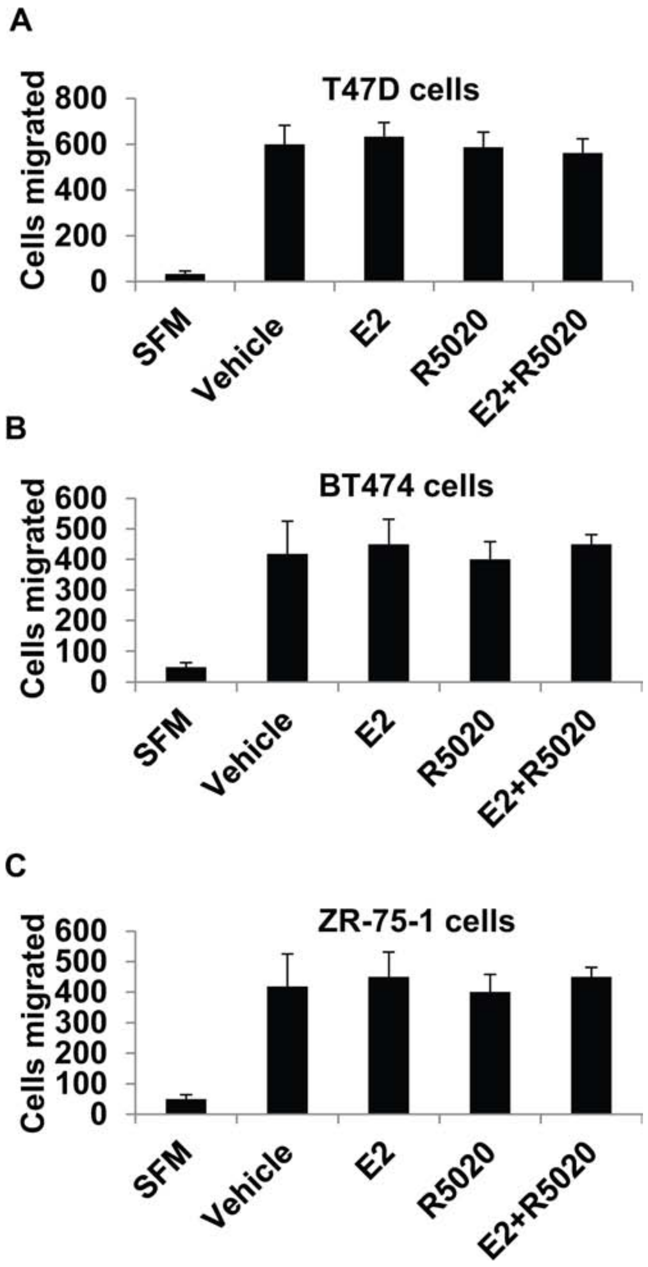

Supplementary Figure S2: Absence of hormonal control of migration capacity of ER+/PR+ model cells. Hormone depleted T47D cells **Panel A.** BT474 cells **Panel B.** and ZR-75–1 cells **Panel C.** at 30% confluence were treated with vehicle, E_2 (1 nM), R5020 (1 nM), or E_2 (1 nM) plus R5020 (1 nM) for 48 h. Cells were trypsinized and subjected to transwell migration assay (i.e., in the absence of matrigel) with vehicle or the appropriate concentration of hormone present in the top and bottom chambers, as described under Materials and Methods. In the negative control, serum free media (SFM) was used instead of the FBS chemoattractant. Values are represented as average numbers of cells migrated from experimental triplicates and the error bars represent standard deviation. One-way ANOVA was performed and there was no significant difference between the treatment groups.

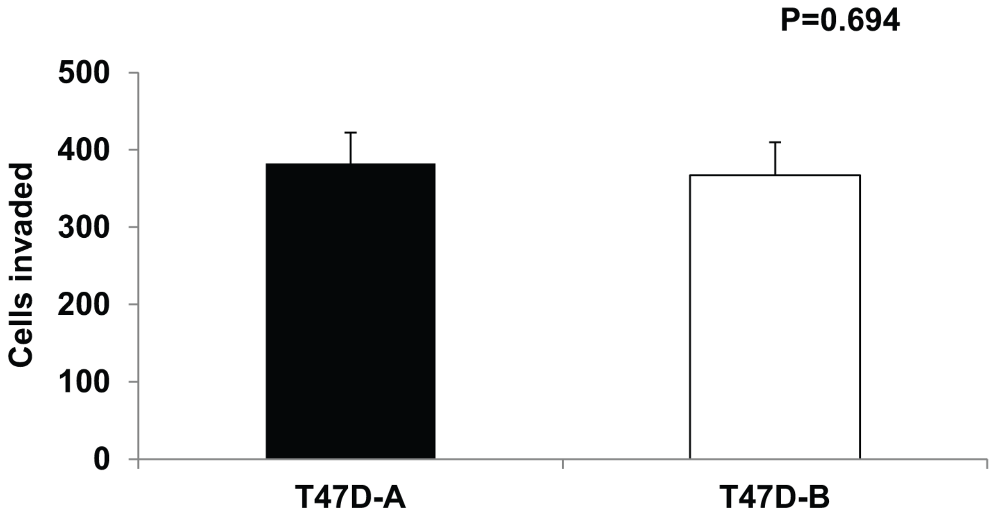

Supplementary Figure S3: Invasive capacity of the isogenic T47D-A vs. T47D-B cell lines. T47D-A and T47D-B cells at 30% confluence were incubated in hormone-depleted media for 96 hours. Cells were trypsinized and subjected to the matrigel transwell invasion assay as described under Materials and Methods. Values are represented as average number of cells invaded from experimental triplicates and the error bars represent standard deviation.

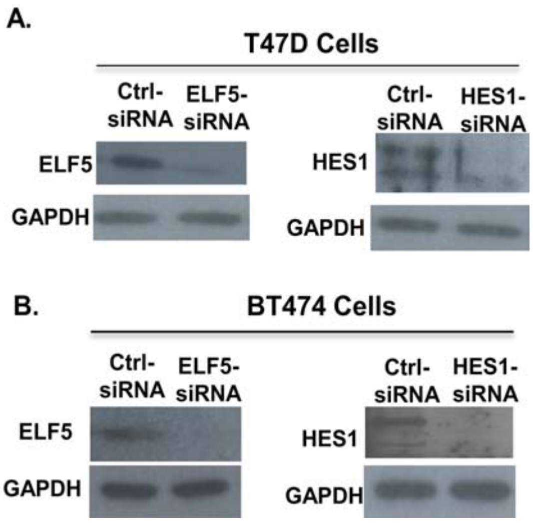

Supplementary Figure S4: Western blots showing knockdown of HES1 and ELF5 using siRNA. This is an extension of the experiment described in Figure 8. Hormone-depleted T47D cells (Panel A) and BT474 (Panel B) were transfected with control siRNA, HES1 siRNA or ELF5 siRNA. After 72 hours, cell lysates were obtained and subjected to western blot analysis, probing for ELF5 and HES1. GAPDH was probed as a loading control.

Supplementary Tables S1–13. mRNA profiling of estrogen (1nM) and progesterone (1nM) regulated genes in cells expressing ER and either PR-A or PR-B. T47D-A and T47D-B cells were depleted of hormone for 48 h as described above. Cells were then either treated with vehicle, 1nM E2, 1nM R5020, or 1nM E2 + 1nM R5020 for 48 h. The samples were analyzed using the HumanHT-12 v4 Expression BeadChip with the Illumina HiScan System. A total of 47,000 probes were used to analyze the transcriptome expression for each treatment group. Expression values were normalized using quantile-normalization, with background subtraction. Log transformation to the base of 2, followed by one way ANOVA was used to determine error. The differentially expressed genes were identified by comparing E2 treatment with vehicle treatment, R5020 treatment with vehicle treatment and E2 treatment with E2+R5020 treatment (repressed or activated with a fold difference of 1.5 and a p value <0.05). Genes that had activated expression in E2+R5020 treatment but were repressed by E2 treatment were identified. Genes that were activated in E2 treatment but repressed by E2+R5020 were also identified.
